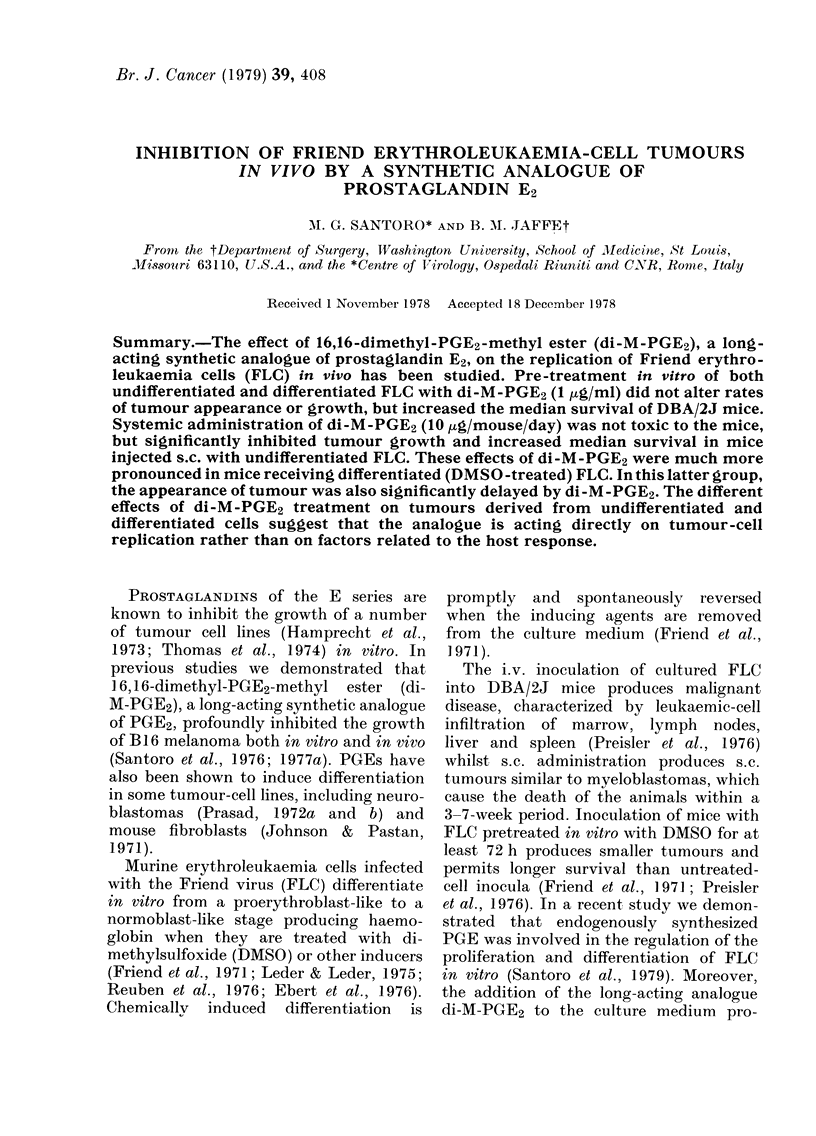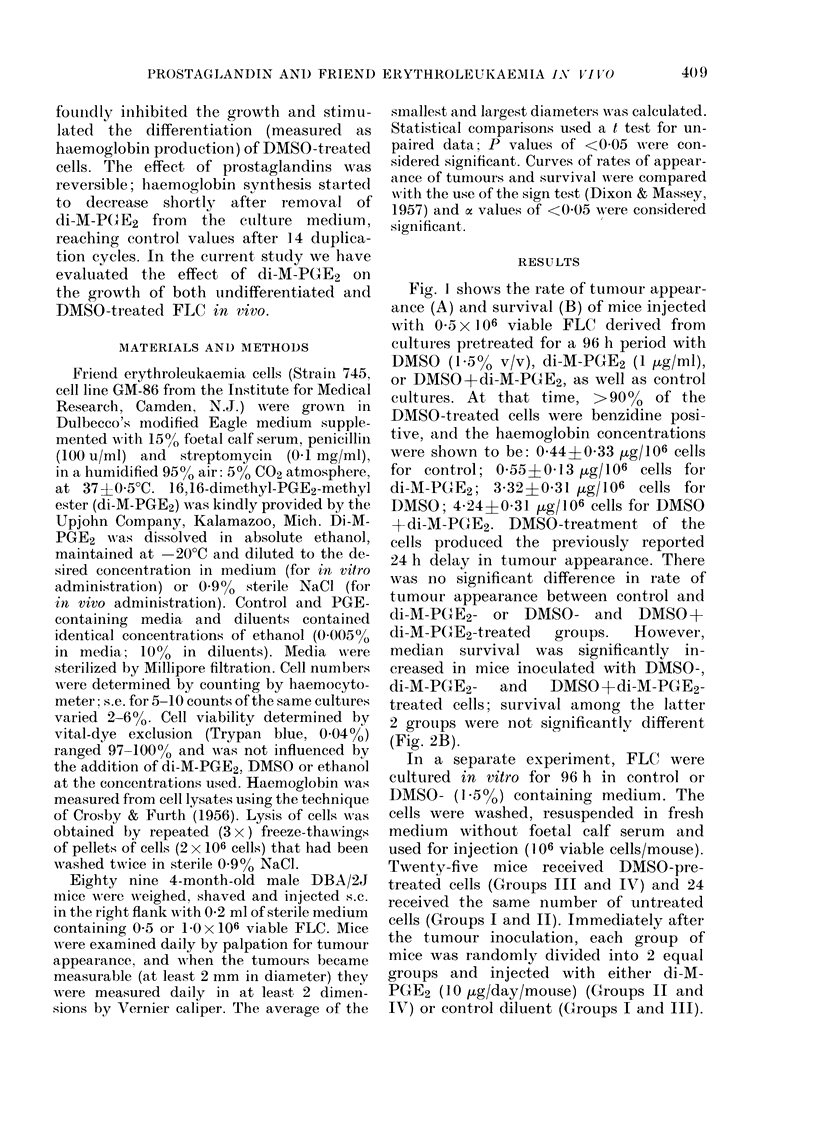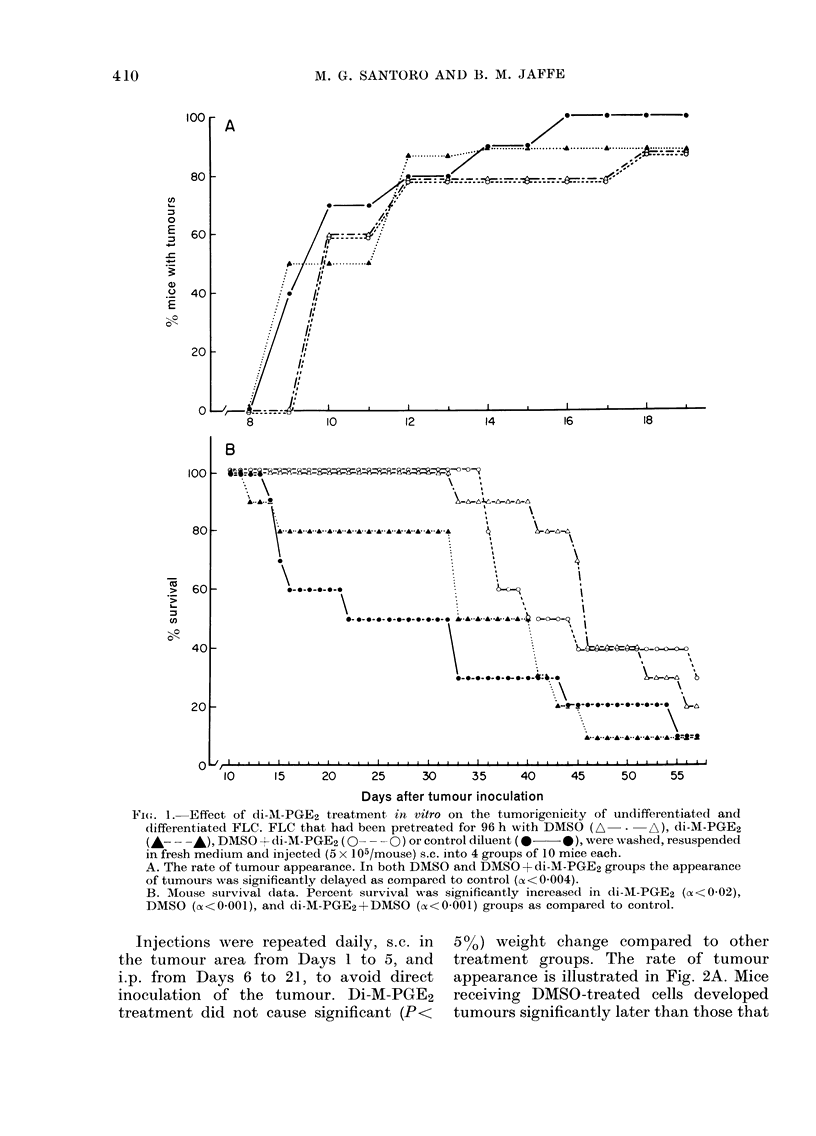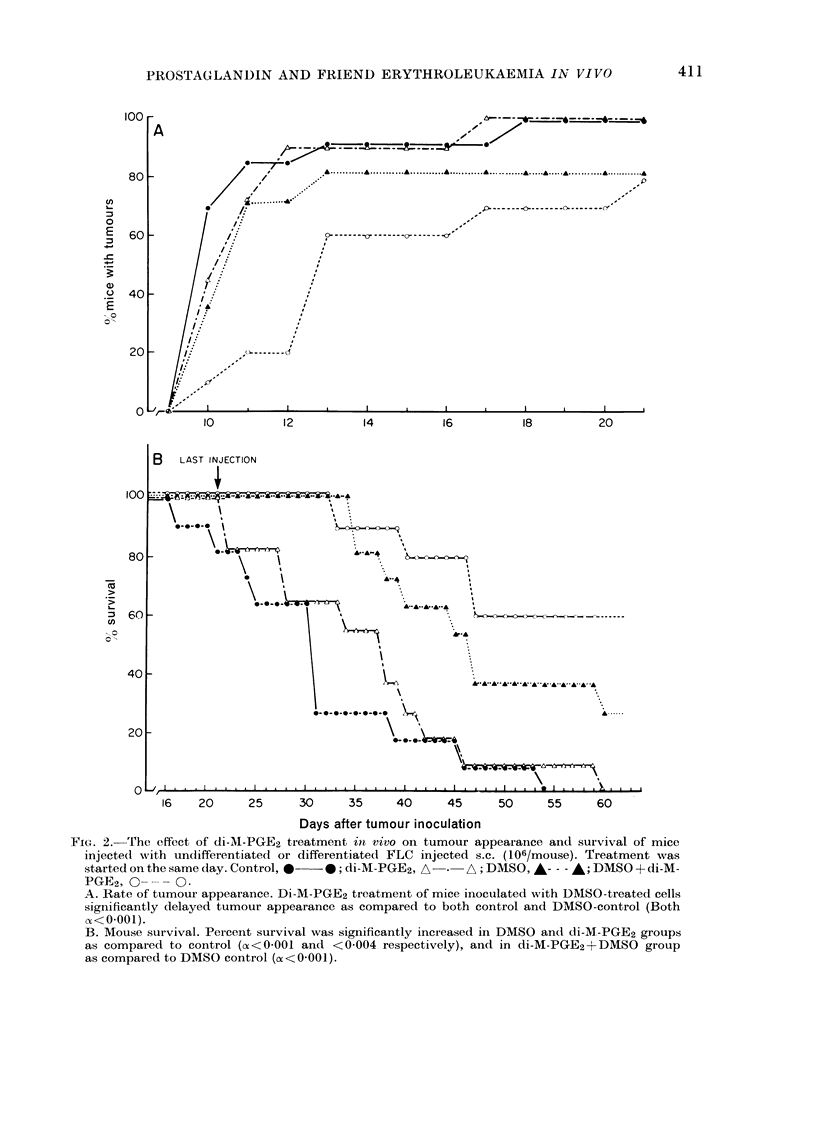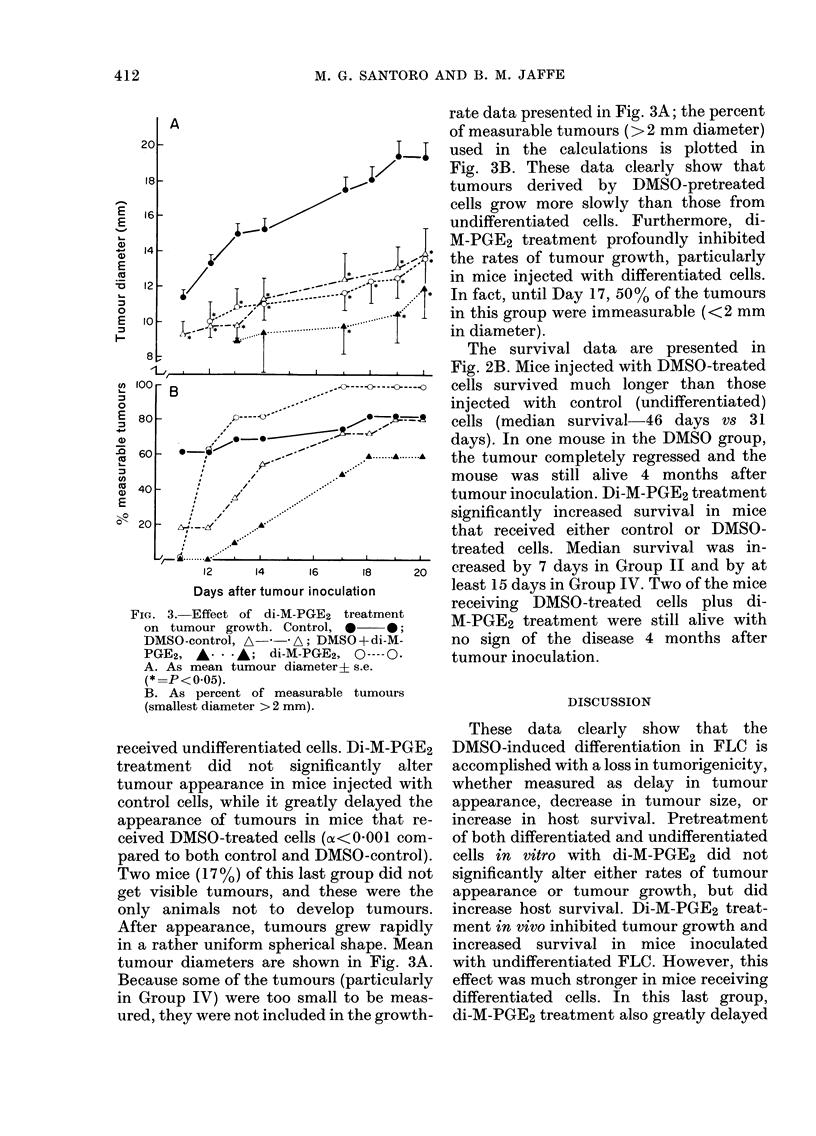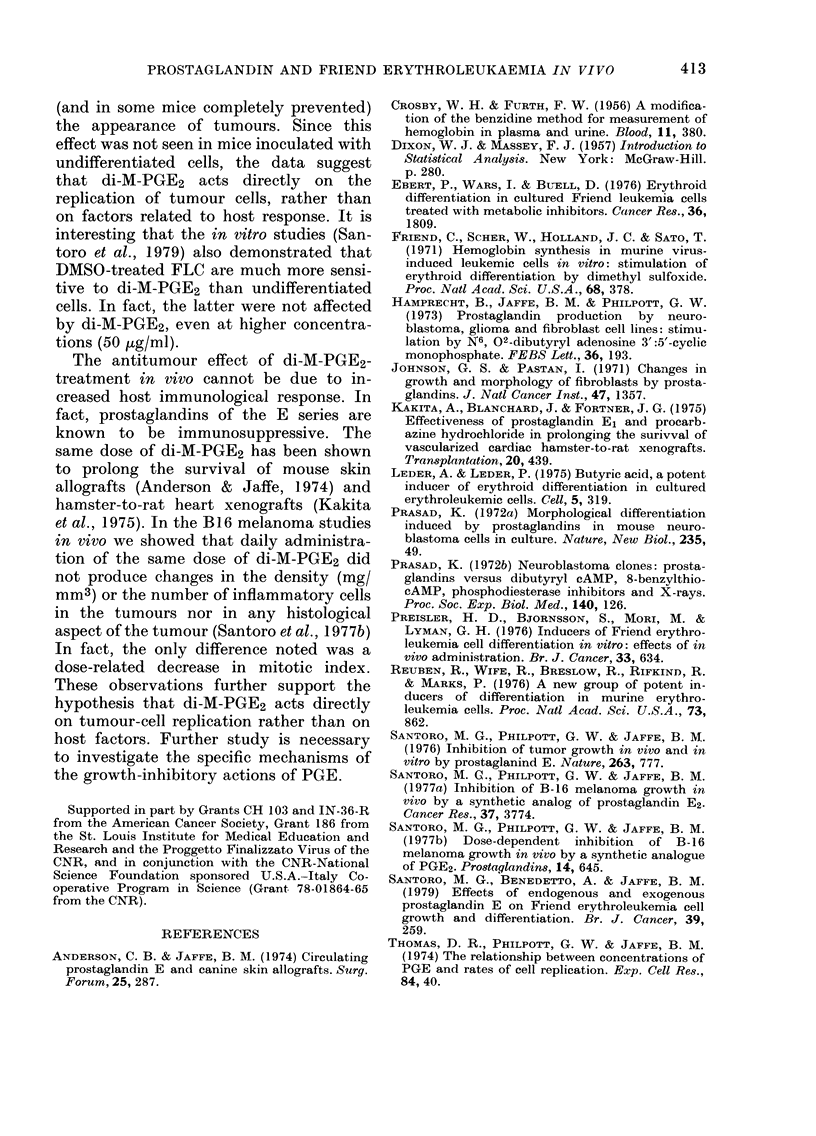# Inhibition of Friend erythroleukaemia-cell tumours in vivo by a synthetic analogue of prostaglandin E2.

**DOI:** 10.1038/bjc.1979.73

**Published:** 1979-04

**Authors:** M. G. Santoro, B. M. Jaffe

## Abstract

The effect of 16,16-dimethyl-PGE2-methyl ester (di-M-PGE2), a long-acting synthetic analogue of prostaglandin E2, on the replication of Friend erythroleukaemia cells (FLC) in vivo has been studied. Pre-treatment in vitro of both undifferentiated and differentiated FLC with di-M-PGE2 (1 microgram/ml) did not alter rates of tumour appearance or growth, but increased the median survival of DBA/2J mice. Systemic administration of di-M-PGE2 (10 microgram/mouse/day) was not toxic to the mice, but significantly inhibited tumour growth and increased median survival in mice injected s.c. with undifferentiated FLC. These effects of di-M-PGE2 were much more pronounced in mice receiving differentiated (DMSO-treated) FLC. In this latter group, the appearance of tumour was also significantly delayed by di-M-PGE2. The different effects of di-M-PGE2 treatment on tumours derived from undifferentiated and differentiated cells suggest that the analogue is acting directly on tumour-cell replication rather than on factors related to the host response.


*Br. J. Cancer* (1979) **39**, 408

### INHIBITION OF FRIEND ERYTHROLEUKAEMIA-CELL TUMOURS *IN VIVO* BY A SYNTHETIC ANALOGUE OF PROSTAGLANDIN $E_2$

M. G. SANTORO* AND B. M. JAFFE†

*From the †Department of Surgery, Washington University, School of Medicine, St Louis, Missouri 63110, U.S.A., and the *Centre of Virology, Ospedali Riuniti and CNR, Rome, Italy*

Received 1 November 1978 Accepted 18 December 1978

**Summary.—The effect of 16,16-dimethyl-PGE$_2$-methyl ester (di-M-PGE$_2$), a long-acting synthetic analogue of prostaglandin E$_2$, on the replication of Friend erythroleukaemia cells (FLC) *in vivo* has been studied. Pre-treatment *in vitro* of both undifferentiated and differentiated FLC with di-M-PGE$_2$ (1 $\mu$g/ml) did not alter rates of tumour appearance or growth, but increased the median survival of DBA/2J mice. Systemic administration of di-M-PGE$_2$ (10 $\mu$g/mouse/day) was not toxic to the mice, but significantly inhibited tumour growth and increased median survival in mice injected s.c. with undifferentiated FLC. These effects of di-M-PGE$_2$ were much more pronounced in mice receiving differentiated (DMSO-treated) FLC. In this latter group, the appearance of tumour was also significantly delayed by di-M-PGE$_2$. The different effects of di-M-PGE$_2$ treatment on tumours derived from undifferentiated and differentiated cells suggest that the analogue is acting directly on tumour-cell replication rather than on factors related to the host response.**

PROSTAGLANDINS of the E series are known to inhibit the growth of a number of tumour cell lines (Hamprecht *et al.*, 1973; Thomas *et al.*, 1974) *in vitro*. In previous studies we demonstrated that 16,16-dimethyl-PGE$_2$-methyl ester (di-M-PGE$_2$), a long-acting synthetic analogue of PGE$_2$, profoundly inhibited the growth of B16 melanoma both *in vitro* and *in vivo* (Santoro *et al.*, 1976; 1977*a*). PGEs have also been shown to induce differentiation in some tumour-cell lines, including neuroblastomas (Prasad, 1972*a* and *b*) and mouse fibroblasts (Johnson & Pastan, 1971).

Murine erythroleukaemia cells infected with the Friend virus (FLC) differentiate *in vitro* from a proerythroblast-like to a normoblast-like stage producing haemoglobin when they are treated with dimethylsulfoxide (DMSO) or other inducers (Friend *et al.*, 1971; Leder & Leder, 1975; Reuben *et al.*, 1976; Ebert *et al.*, 1976). Chemically induced differentiation is promptly and spontaneously reversed when the inducing agents are removed from the culture medium (Friend *et al.*, 1971).

The i.v. inoculation of cultured FLC into DBA/2J mice produces malignant disease, characterized by leukaemic-cell infiltration of marrow, lymph nodes, liver and spleen (Preisler *et al.*, 1976) whilst s.c. administration produces s.c. tumours similar to myeloblastomas, which cause the death of the animals within a 3–7-week period. Inoculation of mice with FLC pretreated *in vitro* with DMSO for at least 72 h produces smaller tumours and permits longer survival than untreated-cell inocula (Friend *et al.*, 1971; Preisler *et al.*, 1976). In a recent study we demonstrated that endogenously synthesized PGE was involved in the regulation of the proliferation and differentiation of FLC *in vitro* (Santoro *et al.*, 1979). Moreover, the addition of the long-acting analogue di-M-PGE$_2$ to the culture medium profoundly inhibited the growth and stimulated the differentiation (measured as haemoglobin production) of DMSO-treated cells. The effect of prostaglandins was reversible; haemoglobin synthesis started to decrease shortly after removal of di-M-$PGE_2$ from the culture medium, reaching control values after 14 duplication cycles. In the current study we have evaluated the effect of di-M-$PGE_2$ on the growth of both undifferentiated and DMSO-treated FLC *in vivo*.

### MATERIALS AND METHODS

Friend erythroleukaemia cells (Strain 745, cell line GM-86 from the Institute for Medical Research, Camden, N.J.) were grown in Dulbecco's modified Eagle medium supplemented with 15% foetal calf serum, penicillin (100 u/ml) and streptomycin (0·1 mg/ml), in a humidified 95% air: 5% $CO_2$ atmosphere, at 37±0·5°C. 16,16-dimethyl-$PGE_2$-methyl ester (di-M-$PGE_2$) was kindly provided by the Upjohn Company, Kalamazoo, Mich. Di-M-$PGE_2$ was dissolved in absolute ethanol, maintained at −20°C and diluted to the desired concentration in medium (for *in vitro* administration) or 0·9% sterile NaCl (for *in vivo* administration). Control and PGE-containing media and diluents contained identical concentrations of ethanol (0·005% in media; 10% in diluents). Media were sterilized by Millipore filtration. Cell numbers were determined by counting by haemocytometer; s.e. for 5–10 counts of the same cultures varied 2–6%. Cell viability determined by vital-dye exclusion (Trypan blue, 0·04%) ranged 97–100% and was not influenced by the addition of di-M-$PGE_2$, DMSO or ethanol at the concentrations used. Haemoglobin was measured from cell lysates using the technique of Crosby & Furth (1956). Lysis of cells was obtained by repeated (3×) freeze-thawings of pellets of cells ($2\times10^6$ cells) that had been washed twice in sterile 0·9% NaCl.

Eighty nine 4-month-old male DBA/2J mice were weighed, shaved and injected s.c. in the right flank with 0·2 ml of sterile medium containing 0·5 or $1{\cdot}0\times10^6$ viable FLC. Mice were examined daily by palpation for tumour appearance, and when the tumours became measurable (at least 2 mm in diameter) they were measured daily in at least 2 dimensions by Vernier caliper. The average of the smallest and largest diameters was calculated. Statistical comparisons used a $t$ test for unpaired data; $P$ values of $<0{\cdot}05$ were considered significant. Curves of rates of appearance of tumours and survival were compared with the use of the sign test (Dixon & Massey, 1957) and $\alpha$ values of $<0{\cdot}05$ were considered significant.

### RESULTS

Fig. 1 shows the rate of tumour appearance (A) and survival (B) of mice injected with $0{\cdot}5\times10^6$ viable FLC derived from cultures pretreated for a 96 h period with DMSO (1·5% v/v), di-M-$PGE_2$ (1 $\mu$g/ml), or DMSO+di-M-$PGE_2$, as well as control cultures. At that time, >90% of the DMSO-treated cells were benzidine positive, and the haemoglobin concentrations were shown to be: 0·44±0·33 $\mu g/10^6$ cells for control; 0·55±0·13 $\mu g/10^6$ cells for di-M-$PGE_2$; 3·32±0·31 $\mu g/10^6$ cells for DMSO; 4·24±0·31 $\mu g/10^6$ cells for DMSO +di-M-$PGE_2$. DMSO-treatment of the cells produced the previously reported 24 h delay in tumour appearance. There was no significant difference in rate of tumour appearance between control and di-M-$PGE_2$- or DMSO- and DMSO+di-M-$PGE_2$-treated groups. However, median survival was significantly increased in mice inoculated with DMSO-, di-M-$PGE_2$- and DMSO+di-M-$PGE_2$-treated cells; survival among the latter 2 groups were not significantly different (Fig. 2B).

In a separate experiment, FLC were cultured *in vitro* for 96 h in control or DMSO- (1·5%) containing medium. The cells were washed, resuspended in fresh medium without foetal calf serum and used for injection ($10^6$ viable cells/mouse). Twenty-five mice received DMSO-pretreated cells (Groups III and IV) and 24 received the same number of untreated cells (Groups I and II). Immediately after the tumour inoculation, each group of mice was randomly divided into 2 equal groups and injected with either di-M-$PGE_2$ (10 $\mu$g/day/mouse) (Groups II and IV) or control diluent (Groups I and III).

FIG. 1.—Effect of di-M-$PGE_2$ treatment *in vitro* on the tumorigenicity of undifferentiated and differentiated FLC. FLC that had been pretreated for 96 h with DMSO (△— · —△), di-M-$PGE_2$ (▲- - -▲), DMSO + di-M-$PGE_2$ (○- - -○) or control diluent (●——●), were washed, resuspended in fresh medium and injected ($5 \times 10^5$/mouse) s.c. into 4 groups of 10 mice each.

A. The rate of tumour appearance. In both DMSO and DMSO+di-M-$PGE_2$ groups the appearance of tumours was significantly delayed as compared to control ($\alpha < 0{\cdot}004$).

B. Mouse survival data. Percent survival was significantly increased in di-M-$PGE_2$ ($\alpha < 0{\cdot}02$), DMSO ($\alpha < 0{\cdot}001$), and di-M-$PGE_2$+DMSO ($\alpha < 0{\cdot}001$) groups as compared to control.

Injections were repeated daily, s.c. in the tumour area from Days 1 to 5, and i.p. from Days 6 to 21, to avoid direct inoculation of the tumour. Di-M-$PGE_2$ treatment did not cause significant ($P <$ 5%) weight change compared to other treatment groups. The rate of tumour appearance is illustrated in Fig. 2A. Mice receiving DMSO-treated cells developed tumours significantly later than those that

FIG. 2.—The effect of di-M-PGE2 treatment *in vivo* on tumour appearance and survival of mice injected with undifferentiated or differentiated FLC injected s.c. ($10^6$/mouse). Treatment was started on the same day. Control, ●——●; di-M-PGE2, △—·—△; DMSO, ▲- - -▲; DMSO+di-M-PGE2, ○– – – ○.

A. Rate of tumour appearance. Di-M-PGE2 treatment of mice inoculated with DMSO-treated cells significantly delayed tumour appearance as compared to both control and DMSO-control (Both $\alpha < 0{\cdot}001$).

B. Mouse survival. Percent survival was significantly increased in DMSO and di-M-PGE2 groups as compared to control ($\alpha < 0{\cdot}001$ and $< 0{\cdot}004$ respectively), and in di-M-PGE2+DMSO group as compared to DMSO control ($\alpha < 0{\cdot}001$).

FIG. 3.—Effect of di-M-PGE2 treatment on tumour growth. Control, ●——●; DMSO-control, △—·—·△; DMSO+di-M-PGE2, ▲· · ·▲; di-M-PGE2, ○----○. A. As mean tumour diameter ± s.e. (* = $P < 0.05$). B. As percent of measurable tumours (smallest diameter > 2 mm).

received undifferentiated cells. Di-M-PGE2 treatment did not significantly alter tumour appearance in mice injected with control cells, while it greatly delayed the appearance of tumours in mice that received DMSO-treated cells ($\alpha < 0.001$ compared to both control and DMSO-control). Two mice (17%) of this last group did not get visible tumours, and these were the only animals not to develop tumours. After appearance, tumours grew rapidly in a rather uniform spherical shape. Mean tumour diameters are shown in Fig. 3A. Because some of the tumours (particularly in Group IV) were too small to be measured, they were not included in the growth-rate data presented in Fig. 3A; the percent of measurable tumours (>2 mm diameter) used in the calculations is plotted in Fig. 3B. These data clearly show that tumours derived by DMSO-pretreated cells grow more slowly than those from undifferentiated cells. Furthermore, di-M-PGE2 treatment profoundly inhibited the rates of tumour growth, particularly in mice injected with differentiated cells. In fact, until Day 17, 50% of the tumours in this group were immeasurable (<2 mm in diameter).

The survival data are presented in Fig. 2B. Mice injected with DMSO-treated cells survived much longer than those injected with control (undifferentiated) cells (median survival—46 days *vs* 31 days). In one mouse in the DMSO group, the tumour completely regressed and the mouse was still alive 4 months after tumour inoculation. Di-M-PGE2 treatment significantly increased survival in mice that received either control or DMSO-treated cells. Median survival was increased by 7 days in Group II and by at least 15 days in Group IV. Two of the mice receiving DMSO-treated cells plus di-M-PGE2 treatment were still alive with no sign of the disease 4 months after tumour inoculation.

### DISCUSSION

These data clearly show that the DMSO-induced differentiation in FLC is accomplished with a loss in tumorigenicity, whether measured as delay in tumour appearance, decrease in tumour size, or increase in host survival. Pretreatment of both differentiated and undifferentiated cells *in vitro* with di-M-PGE2 did not significantly alter either rates of tumour appearance or tumour growth, but did increase host survival. Di-M-PGE2 treatment *in vivo* inhibited tumour growth and increased survival in mice inoculated with undifferentiated FLC. However, this effect was much stronger in mice receiving differentiated cells. In this last group, di-M-PGE2 treatment also greatly delayed

(and in some mice completely prevented) the appearance of tumours. Since this effect was not seen in mice inoculated with undifferentiated cells, the data suggest that di-M-$PGE_2$ acts directly on the replication of tumour cells, rather than on factors related to host response. It is interesting that the *in vitro* studies (Santoro *et al.*, 1979) also demonstrated that DMSO-treated FLC are much more sensitive to di-M-$PGE_2$ than undifferentiated cells. In fact, the latter were not affected by di-M-$PGE_2$, even at higher concentrations (50 $\mu$g/ml).

The antitumour effect of di-M-$PGE_2$-treatment *in vivo* cannot be due to increased host immunological response. In fact, prostaglandins of the E series are known to be immunosuppressive. The same dose of di-M-$PGE_2$ has been shown to prolong the survival of mouse skin allografts (Anderson & Jaffe, 1974) and hamster-to-rat heart xenografts (Kakita *et al.*, 1975). In the B16 melanoma studies *in vivo* we showed that daily administration of the same dose of di-M-$PGE_2$ did not produce changes in the density (mg/$mm^3$) or the number of inflammatory cells in the tumours nor in any histological aspect of the tumour (Santoro *et al.*, 1977*b*) In fact, the only difference noted was a dose-related decrease in mitotic index. These observations further support the hypothesis that di-M-$PGE_2$ acts directly on tumour-cell replication rather than on host factors. Further study is necessary to investigate the specific mechanisms of the growth-inhibitory actions of PGE.

Supported in part by Grants CH 103 and IN-36-R from the American Cancer Society, Grant 186 from the St. Louis Institute for Medical Education and Research and the Proggetto Finalizzato Virus of the CNR, and in conjunction with the CNR-National Science Foundation sponsored U.S.A.–Italy Cooperative Program in Science (Grant 78-01864-65 from the CNR).

### REFERENCES

ANDERSON, C. B. & JAFFE, B. M. (1974) Circulating prostaglandin E and canine skin allografts. *Surg. Forum*, **25**, 287.

CROSBY, W. H. & FURTH, F. W. (1956) A modification of the benzidine method for measurement of hemoglobin in plasma and urine. *Blood*, **11**, 380.

DIXON, W. J. & MASSEY, F. J. (1957) *Introduction to Statistical Analysis.* New York: McGraw-Hill. p. 280.

EBERT, P., WARS, I. & BUELL, D. (1976) Erythroid differentiation in cultured Friend leukemia cells treated with metabolic inhibitors. *Cancer Res.*, **36**, 1809.

FRIEND, C., SCHER, W., HOLLAND, J. C. & SATO, T. (1971) Hemoglobin synthesis in murine virus-induced leukemic cells *in vitro*: stimulation of erythroid differentiation by dimethyl sulfoxide. *Proc. Natl Acad. Sci. U.S.A.*, **68**, 378.

HAMPRECHT, B., JAFFE, B. M. & PHILPOTT, G. W. (1973) Prostaglandin production by neuroblastoma, glioma and fibroblast cell lines: stimulation by $N^6$, $O^2$-dibutyryl adenosine 3′:5′-cyclic monophosphate. *FEBS Lett.*, **36**, 193.

JOHNSON, G. S. & PASTAN, I. (1971) Changes in growth and morphology of fibroblasts by prostaglandins. *J. Natl Cancer Inst.*, **47**, 1357.

KAKITA, A., BLANCHARD, J. & FORTNER, J. G. (1975) Effectiveness of prostaglandin $E_1$ and procarbazine hydrochloride in prolonging the surivval of vascularized cardiac hamster-to-rat xenografts. *Transplantation*, **20**, 439.

LEDER, A. & LEDER, P. (1975) Butyric acid, a potent inducer of erythroid differentiation in cultured erythroleukemic cells. *Cell*, **5**, 319.

PRASAD, K. (1972*a*) Morphological differentiation induced by prostaglandins in mouse neuroblastoma cells in culture. *Nature, New Biol.*, **235**, 49.

PRASAD, K. (1972*b*) Neuroblastoma clones: prostaglandins versus dibutyryl cAMP, 8-benzylthio-cAMP, phosphodiesterase inhibitors and X-rays. *Proc. Soc. Exp. Biol. Med.*, **140**, 126.

PREISLER, H. D., BJORNSSON, S., MORI, M. & LYMAN, G. H. (1976) Inducers of Friend erythroleukemia cell differentiation *in vitro*: effects of *in vivo* administration. *Br. J. Cancer*, **33**, 634.

REUBEN, R., WIFE, R., BRESLOW, R., RIFKIND, R. & MARKS, P. (1976) A new group of potent inducers of differentiation in murine erythroleukemia cells. *Proc. Natl Acad. Sci. U.S.A.*, **73**, 862.

SANTORO, M. G., PHILPOTT, G. W. & JAFFE, B. M. (1976) Inhibition of tumor growth *in vivo* and *in vitro* by prostaglanind E. *Nature*, **263**, 777.

SANTORO, M. G., PHILPOTT, G. W. & JAFFE, B. M. (1977*a*) Inhibition of B-16 melanoma growth *in vivo* by a synthetic analog of prostaglandin $E_2$. *Cancer Res.*, **37**, 3774.

SANTORO, M. G., PHILPOTT, G. W. & JAFFE, B. M. (1977b) Dose-dependent inhibition of B-16 melanoma growth *in vivo* by a synthetic analogue of $PGE_2$. *Prostaglandins*, **14**, 645.

SANTORO, M. G., BENEDETTO, A. & JAFFE, B. M. (1979) Effects of endogenous and exogenous prostaglandin E on Friend erythroleukemia cell growth and differentiation. *Br. J. Cancer*, **39**, 259.

THOMAS, D. R., PHILPOTT, G. W. & JAFFE, B. M. (1974) The relationship between concentrations of PGE and rates of cell replication. *Exp. Cell Res.*, **84**, 40.